# Efficacy and safety of vitamin D supplementation on psoriasis: A systematic review and meta-analysis

**DOI:** 10.1371/journal.pone.0294239

**Published:** 2023-11-15

**Authors:** Qianqian Dai, Yanfeng Zhang, Qian Liu, Chijin Zhang

**Affiliations:** 1 Tianjin University of Traditional Chinese Medicine, Tianjin, China; 2 Department of Dermatology, Shushan TCM Clinic, Anhui Xin’an TCM Medical Service Co., LTD., Hefei, China; 3 Department of Dermatology, Tangshan Fengnan Hospital of Traditional Chinese Medicine, Tangshan, China; 4 Anhui University of Traditional Chinese Medicine, Hefei, China; 5 Department of Dermatology, The First Affiliated Hospital of Tianjin University of Traditional Chinese Medicine, Tianjin, China; University of Bahrain, BAHRAIN

## Abstract

**Objectives:**

Our aim was to analyze the results of published randomized controlled trials (RCTs) on vitamin D supplementation for psoriasis in order to explore its effectiveness and safety.

**Patients and methods:**

As of July 7 2023, we conducted a systematic literature search in PubMed, Cochrane, Embase, and Web of Science Core Collection databases. The study outcomes included change values in Psoriasis Area and Severity Index (PASI) (at 3 months, 6 months, and end of follow-up)/Dermatology Life Quality Index (DLQI)/Psoriasis disability index (PDI)/C-reactive protein (CRP), and adverse events.

**Results:**

333 patients from 4 studies were evaluated. Pooled analyses showed no significant effect of DLQI/PDI/CRP change value (P > 0.05) or PASI change value (3 months, end of follow-up; P > 0.05). Sensitivity analyses and statistical tests did not support the results of the PASI change values (6 months, P = 0.05). However, the results of subgroup analyses should not be ignored(supplementation with vitamin D2 or Asia would be more effective; P = 0.03). There were no serious adverse effects, and only a few individuals experienced nausea.

**Conclusions:**

The efficacy and safety of vitamin D supplementation in the treatment of psoriasis remains unremarkable. The search for a new prognostic index that combines clinical and laboratory factors is needed to compensate for the shortcomings of existing measures and provide stronger evidence of validity.

## Introduction

Psoriasis is a chronic inflammatory disease which is immune-mediated and with a complex pathogenesis. Hyperproliferative epidermis with abnormal differentiation usually results in the psoriatic skin lesions. According to the World Health Organization, there are more than 100 million psoriasis patients worldwide [1, 2]. The incidence estimated in adults ranged from 0.51% to 11.43% while in children it ranged from 0% to 1.37%. Approximately 78% to 90% of psoriasis patients are diagnosed as mild to moderate skin lesions [3–5]. Some previous studies have claimed peripheral vitamin D was deficient or insufficient in psoriasis patients, and this may contribute to the development of the disease [6].

Vitamin D exists in two distinct forms, namely ergocalciferol (vitamin D2) and cholecalciferol (vitamin D3). These are both fat-soluble vitamins [7]. Vitamin D2 is synthesized by plants, while vitamin D3 is sourced from animal-derived foods. As many studies reported, Vitamin D not only plays a positive role in regulating bone and calcium homeostasis, but also in immunomodulation. In the realm of dermatology, Vitamin D plays a pivotal role in influencing a multitude of physiological processes within the skin. These processes encompass keratinocyte proliferation, differentiation, apoptosis, in addition to its crucial involvement in maintaining the skin barrier and regulating immunological responses [8]. There are many ways to utilize vitamin D, and the oral approach to vitamin D supplementation remains controversial in its effectiveness [9] due to its role in immune homeostasis [10].

Several systematic reviews and meta-analyses of the efficacy of vitamin D supplementation in psoriasis have noted that supplementation has failed to show a significant effect [11, 12]. However, it is important to note that the effectiveness conclusions of these studies were based solely on the PASI ignoring the shortcomings of the PASI itself and the impact of improvements in patients’ quality of life on effectiveness [13]. Also, the lack of subgroup analysis resulted in unexplored possible potential validity. Therefore, we extended the scope of evaluation indexes (PASI, DLQI, PDI were used) and performed subgroup analysis, and also introduced the association marker CRP, in order to evaluate and validate the efficacy and safety of oral vitamin D supplementation in the treatment of psoriasis from more perspectives.

## Materials and methods

### Literature search

The present study was performed in accord with the PRISMA (Preferred Reporting Items for Systematic Reviews and Meta-Analysis, S1 Table) statement in 2020 [14] and was prospectively registered in the PROSPERO (CRD42023441886). A systematic literature search was conducted by two investigators in July 7 2023 from the four databases of PubMed, Cochrane, Embase, and Web of Science Core Collection. The language was restricted to English. We searched using the following MeSH and freewords: “Psoriasis”, “vitamin D” and “supplementation”. The search strategy is specified in S2 Table. All eligible literatures went through multiple rounds of manual review (at least 2 investigators, whom were all dermatology clinicians) to ensure that any disagreements were eliminated, and that the eligible studies met the needs of present analysis with practical relevance.

### Inclusion and exclusion criteria

The search strategy was constructed according to the PICOS acronym as follows: Participants: adults suffering with any type of psoriasis; Interventions: vitamin D supplementation; Comparison: placebo without vitamin D supplementation; Outcomes: Change of Psoriasis Area and Severity Index (PASI) score/Psoriasis disability index (PDI) score/Dermatology Life Quality Index (DLQI)/C-reactive protein (CRP); Study design: randomized controlled trials. Exclusion criteria: reviews, letters, comments, conference abstracts, case reports, pediatric articles, unpublished and non-English articles.

### Data extraction

The proceedings of data extraction were finished by two independent researchers, and any disagreements in the process were settled via discussion. We extracted the data from included studies considering the following information: first author, published year, country of study, study span, study design, registration number, sample size of each study, gender, age and body mass index (BMI) of participants, types of vitamin D, follow-up time(3 mouths/6 mouths/ end of follow-up), Psoriasis Area and Severity Index (PASI), Psoriasis disability index (PDI), Dermatology Life Quality Index (DLQI), Serum 25(OH)D (25 hydroxyvitamin D), C-reactive protein(CRP).

### Quality assessment

The estimation of methodological quality on included RCTs was conducted by 2 independent researchers following the Cochrane Handbook for Systematic Reviews of Interventions 5.1.0 consisting of seven terms: random sequence generation, allocation concealment, blinding of participants and personnel, blinding of outcome assessment, incomplete outcome data, selective reporting and other sources of bias. Each study aspects was accessed and classified into three levels: low risk, high risk and unclear risk. Studies with more “low risk” bias evaluations were regarded as superior [15]. When disagreements were encountered, the judgment was given by another, higher level researcher.

### Statistical analysis

The analysis of included studies was done by using Review Manager version 5.4.1(Cochrane Collaboration, Oxford, UK). The WMD was used to assess the continuous data, and dichotomous variables was analyzed with RR. All outcome indicators were calculated and given 95% confidence intervals (95% CIs), and the discrepancy in heterogeneity among studies was estimated via inconsistency index (*I*^2^) [16]. Also, we will analyze all data using a random effects model. In addition, possible sources of heterogeneity in our study When the amount of data was greater than 10, funnel plots were drawn and tested using the Egger regression test in Stata version 15.0 statistics. P values < 0.05 were considered statistically significant.

## Results

### Screening results and study characteristics

**Fig 1:**
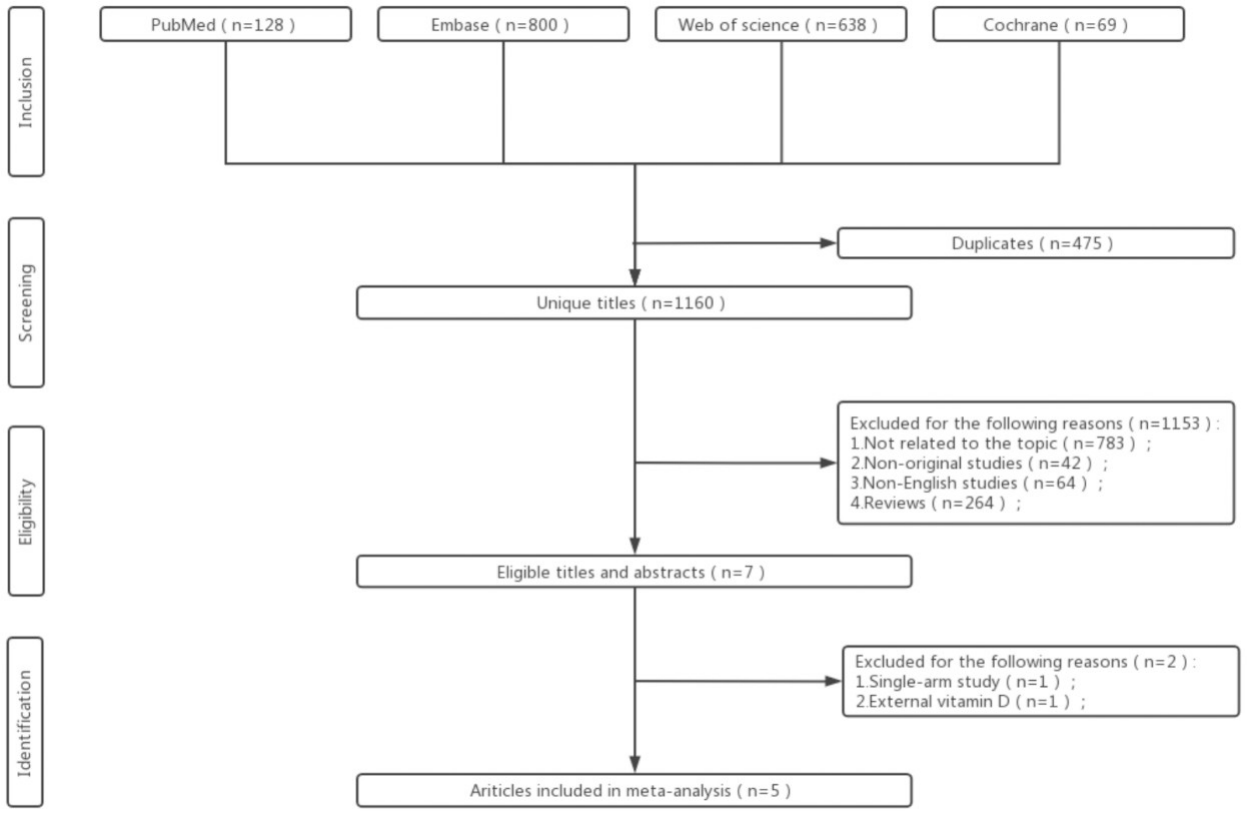
Flowchart of literature screening process.

The literature screening approach and process was performed as shown in Fig 1. Total 1635 publications were searched, comprising PubMed (n = 128), Embase (n = 800), Cochrane (n = 69), and Web of Science (n = 638). After excluding all non-compliant literatures with the inclusion and exclusion criteria, 5 literatures with 333 cases (173 in the experimental group supplemented with vitamin D and 160 in the control group without Vit D supplementation) were finally remained for present study [17–21]. It is important to note that 1 of these 5 studies (El-Hanafy et al) [17] used a methotrexate mixed with vitamin D versus methotrexate regimen, and it will be excluded from the calculations. However, it will be mentioned in the Assessment of Study Quality and Risk of Bias section as well as in the Discussion section because of the information it may suggest. The basic traits of each included study were presented in Table 1, and no statistically significant discrepancy (p > 0.05) was found between the experimental and control groups for Age, Gender, BMI, PASI, Serum 25(OH)D, CRP as shown in Table 2.

**Table 1:** Basic characteristics of included literatures.

Authors	Study period	Country	Study design	Registration number	Types of vitamin D	Patients (n)	Follow-up
						VD/Control	
**Disphanurat 2019**	2016–2017	Thailand	Double-blind RCT	TCTR20180613001	Oral vitamin D2	23/22	6 months
**Ingram 2018**	2012–2013	New Zealand and Australia	Double-blind RCT	12611000648921	Vitamin D3	67/34	12 months
**Jarrett 2018**	2011–2012	New Zealand	Double-blind RCT	ACTRN12611000402943	Vitamin D3 oral capsule	23/42	12 months
**Jenssen 2023**	2017–2019	Norway	Double-blind RCT	NCT03334136	Cholecalciferol	60/62	4 months

VD, vitamin D; RCT, randomized controlled trial

**Table 2:** Demographics and clinical characteristics of included studies.

Outcomes	Studies	No. of patients	WMD or RR	95% CI	P-value	Heterogeneity			
		VD/Control				Chi^2^	df	P-value	*I*^2^ (%)
**Age (years)**	4	173/160	1.78	[-0.58, 4.14]	0.14	3.33	3	0.34	10
**Gender (male)**	4	173/160	1.04	[0.86, 1.25]	0.72	0.87	3	0.83	0
**BMI (kg/m2)**	3	113/98	-0.07	[-1.47, 1.33]	0.93	3.15	2	0.21	37
**PASI**	4	173/160	0.29	[-0.25, 0.83]	0.29	0.08	3	0.99	0
**Serum 25(OH)D**	4	173/160	0.18	[-0.04, 0.40]	0.10	0.96	3	0.81	0
**CRP**	2	90/56	0.89	[-3.02, 4.79]	0.66	6.69	1	0.02	82

BMI, body mass index; PASI, Psoriasis Area and Severity Index; CRP, C-reactive protein; VD, vitamin D; WMD, weighted mean difference; RR, risk ratio; CI, confidence interval.

### Assessment of study quality and risk of bias

**Fig 2:**
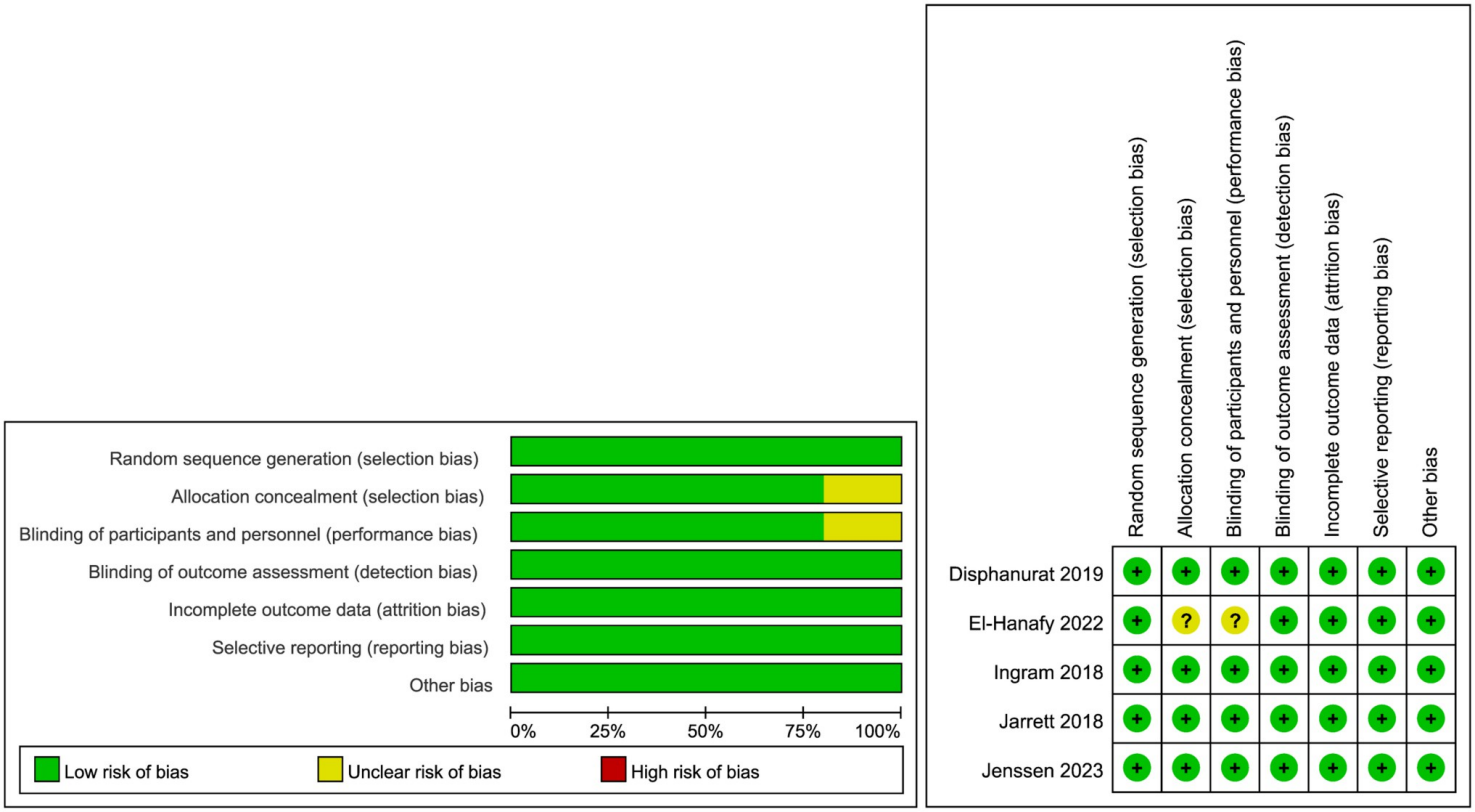
Risk of bias evaluation of included studies.

The quality level of evidence were scored in Fig 2. Four papers were rated as low risk in all evaluation indicators [18–21], and one study was rated as unclear risk of bias in allocation concealmect (selection bias) and blinding of participants/personnel (performance bias) [17].

### Outcomes of meta-analysis

#### Change of psoriasis area and severity index (PASI) (3 mouths)

**Fig 3:**
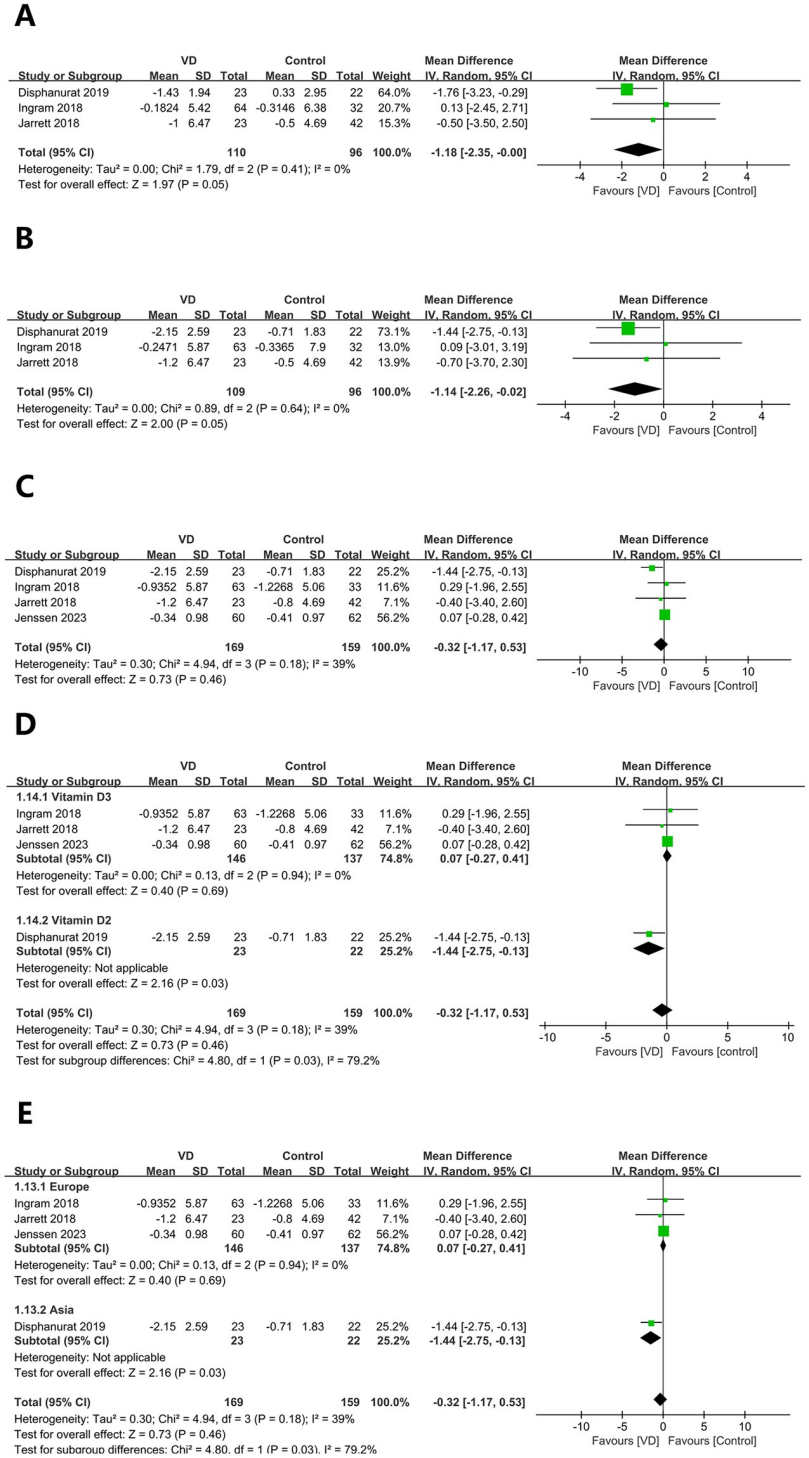
Forest plot of the change in psoriasis area and severity index (PASI):(A)3 mouths, (B) 6 mouths, (C) at the end of follow-up, (D) subgroup of Vitamin D type at the end of follow-up,and (E) subgroup of patient population at the end of follow-up.

A total of 206 patients (110 in the experimental group and 96 in the control group) were included in the analysis. The analysis indicated no significant difference in heterogeneity (*I*^2^ = 0%, p = 0.41) among included studies and the pooled results of random effect model illustrated that vitamin D supplementation for 3 months failed to significantly affect the value of change in PASI (WMD: -1.18; 95% CI: -2.35, 0.00; p = 0.05) (see Fig 3A).

#### Change of psoriasis area and severity index (PASI) (6 mouths)

Total 3 studies with 205 cases (109 were in the experimental group and 96 were in control group) were included. The combined analysis of random effect model showed that vitamin D supplementation for 6 months may significantly affect the value of change in PASI (WMD: -1.14; 95% CI: -2.26, -0.02; p = 0.05) (see Fig 3B). No significant discrepancy in heterogeneity (*I*^2^ = 0%, p = 0.64) nor significant bias in publication.

#### Change of psoriasis area and severity index (PASI) (the end of follow-up)

Included in present analysis were 4 studies with 328 patients (169 were in experimental group and 159 were in palcebo group). The combined analysis revealed that vitamin D supplementation failed to significantly affect the value of change in PASI at the end of follow-up (WMD: -0.32; 95% CI: -1.17, 0.53; p = 0.46)(see Fig 3C). No significant heterogeneity (*I*^2^ = 39%, p = 0.18) nor significant publication bias were observed. Further subgroup analysis was conducted in terms of the type of vitamin D supplementation and the region where the participants were located.

In aspects of the type of vitamin D supplementation, our pooled analysis showed that vitamin D2 supplementation (WMD: -1.44; 95% CI: -2.75, -0.13; p = 0.03) was superior to vitamin D3 (WMD: -0.27; 95% CI: -0.27, 0.41; p = 0.69, no significant difference in heterogeneity: *I*^2^ = 0%, p = 0.94) (see Fig 3D), with significant differences in heterogeneity of the two groups (*I*^2^ = 79.2%, p = 0.03).

In aspects of patients’ location, the pooled results revealed that vitamin D supplementation was superior in Asia (WMD: -1.44; 95% CI: -2.75, -0.13; p = 0.03,) than that in Europe (WMD: 0.07; 95% CI: -0.27, 0.41; p = 0.69, no significant discrepancy in heterogeneity: *I*^2^ = 0%, p = 0.94) (see Fig 3E).

#### Change of psoriasis disability index (PDI)

Of the 4 studies included in the calculations only the change of PDI was provided in the study by Jarrett et al. [20] 65 patients were included (23 in the vitamin D group and 42 in the placebo group). The results showed that the group using vitamin D (-1.4000 + 7.5949) was superior to the placebo group (-0.7000 + 7.9544), but showed no statistical significance(p = 0.73).

#### Change of dermatology life quality index (DLQI)

Of the 4 studies included in the calculations only the study by Jenssen et al. [18] provided values for the change of DLQI and included 122 patients (60 in the vitamin D group and 62 in the placebo group). The results showed that the use of the vitamin D group (-0.59) was superior to the placebo group (0.10). However, the authors concluded that the effect of vitamin D supplementation on DLQI was not significant(p = 0.11).

#### Change of C-reactive protein(CRP)

Of the 4 studies included in the calculations only the study by Disphanurat et al. [21] provided Change of CRP and included 45 patients (23 in the vitamin D group and 22 in the placebo group). The results were not statistically supported(3-mouths p = 0.08,6-mouths p = 0.58), although they showed that at 3 months, the vitamin D group (-3.2482±6.24) was superior to the placebo group (-0.84±3.88); at 6 months, the vitamin D group (-1.03±9.07) was still superior to the placebo group (0.30±3.01).

#### Safety of vitamin D supplementation

Adverse effects of vitamin D supplements were discussed in two of the five studies that participated in the investigation. The study by Ingram et al.[19] found no significant evidence of toxicity and received no reports of adverse effects. In the study by Disphanurat et al [21], at 3-month follow-up, two patients in Vit D supplement group and one case in the placebo group experienced nausea. Accordingly, the study’s personnel concluded that the incidence of adverse reactions to vitamin D supplements for psoriasis is usually low.

### Sensitivity analysis

**Fig 4:**
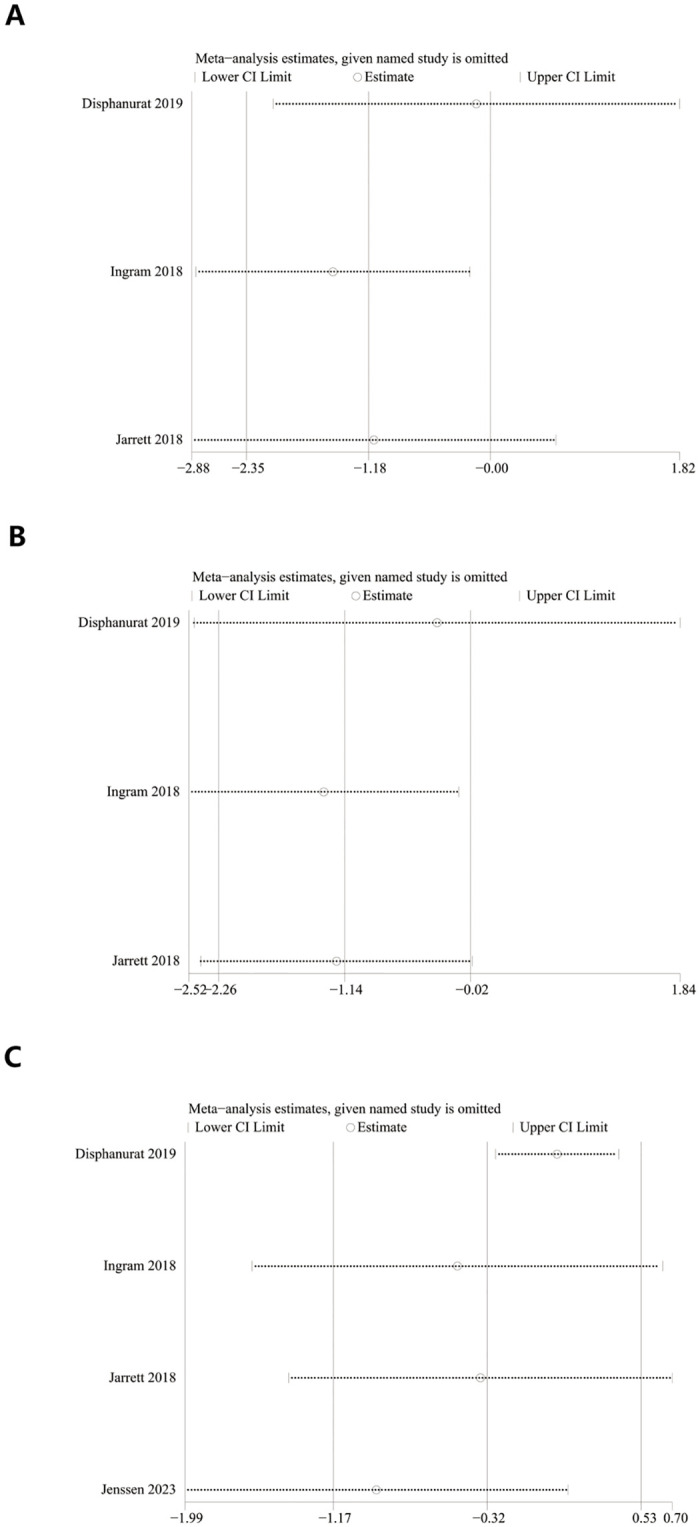
Sensitivity analysis of change in PASI: (A) 3 mouths, (B) 6 mouths, and (C) at the end of follow-up.

We performed a one-way sensitivity analysis comparing changes in PASI at 3 months, 6 months, and the end of follow-up to evaluate the impact of individual study on the composite results. The analysis was calculated through omiting individual studies, and results showed that excluding the 2018 study by Ingarm et al. [19] changed the WMD of PASI at 3 months(see Fig 4A), 6 months(see Fig 4B), but did not change the WMD of PASI at the end of follow-up(see Fig 4C). This result shows that the values of change in PASI at 3 and 6 months are not stable, but at the end of follow-up is robust.

## Discussion

Since vitamin D was discovered and then used for psoriasis in 1985 [22], several landmark studies have been published and provided initial evaluations on this treatment [23–25]. However, as it is controversial whether oral vitamin D supplementation has significant efficacy [26–38], and given that psoriasis is a chronic and complex disease, effective treatment needs to take into account both the physiologic manifestations of the disease and less obvious aspects such as the impact on patients’ quality of life [13]. Therefore, the validity of this study consists of the PASI, which is common for evaluating the physiologic manifestations of the disease, the DLQI, which is common for evaluating the quality of life of patients, and the PDI for psoriasis. At the same time, the study also did subgroup analyses to take into account the potential validity that may exist for population differences and differences in vitamin D types. In addition, to compensate for the lack of sensitivity in selecting indicators, our study introduced CRP as a validation indicator [29].

Three of the publicly available RCTs failed to produce significant efficacy [18–20], and these studies that were found to be ineffective all showed small changes that were thought to have been influenced by other confounding factors rather than vitamin D supplementation, and it should also be mentioned that the indicators of assessment appeared to be insufficiently sensitive to such small changes, also confusing the team in conducting the evaluations. Interestingly, another study [21] that included mildly symptomatic cases and used higher doses of vitamin D supplementation came to the opposite conclusion, which may be that small changes were amplified to render the results significantly useful, and this discrepancy in results also hints at the potential validity of such small changes. At the same time, it should not be overlooked that although the study by El-Hanafy et al [17] was based on methotrexate combined with vitamin D supplementation versus methotrexate alone, the conclusion also supports the effectiveness of vitamin D supplementation. Of course, this conclusion is also suggesting that vitamin D supplementation might yield greater utility when used as an adjunctive treatment. What also strikes one as coincidental is that, regardless of the significant or non-significant conclusions given by these studies, the studies all agree that subgroup analyses should be conducted to seek possible potential effectiveness, and all agree that higher levels of 25(OH)D are effective in the treatment of psoriasis. Apparently, there are three areas worth noting: dose, subgroup analysis, and evaluation metrics.

First, there are two central issues with dosage. One is the effect on intestinal calcium absorption and the calcium homeostasis system, which may lead to hypercalcemia or hypercalciuria [28, 30]. According to the recommendations of the Institute of Medicine and Endocrinology, the daily dose of vitamin D supplementation should be 4,000–10,000 IU/day [31, 32]. The minimum supplementation of 2857 IU/day and the maximum of 4285 IU/day in the studies we included were calculated to be within the recommended range. And in terms of the statistics, there were no serious adverse effects, but nausea was observed in a small number of cases. Of course, there are also studies further indicating that vitamin D supplementation of up to 10,000 IU/day (almost as much as the skin produces on its own) has no association with any harmful effects [33]. On the other hand, there is the dose that produces a significant effect, and we note that Ingram et al. reported an increase in 25(OH)D levels from 24.8 ng/mL to 41.2 ng/mL after 200,000 IU followed by 100,000 IU/month of vitamin D3 [19], but showed ineffective results; Jenssen et al. [18] also described similar results (from 15.1 ng/mL to 29.7 ng/mL); in contrast, Disphanurat et al. showed that 60,000 IU of vitamin D2 every two weeks improved PASI scores, but 25(OH)D levels only increased from 24.77 ng/mL to 27.39 ng/mL [21]. This puzzling situation suggests that the heterogeneity in the dose of vitamin D supplements of significant utility is so high that it is almost impossible to draw definitive conclusions. Therefore, although the data from established studies do not suggest that safety is in question, based on the fact that the dose still needs to be explored and the well-known close relationship between it and safety, we believe that the safety of vitamin D supplementation in the treatment of psoriasis remains unclear.

Second, we conducted a subgroup analysis to look for more possible effectiveness related to two factors: vitamin D type and patient population. Although overall statistical analyses of subgroups for PASI (change at the end of follow-up) values did not show significant evidence of effectiveness, subgroup analyses suggest that supplementation with vitamin D2 may be superior to vitamin D3 and may be more effective in Asian populations than in European populations. Differences between different types of vitamin D and overall may need to be analyzed from a historical perspective.For a long time, vitamin D2 and D3, as two different forms of vitamin D, were considered equivalent and interchangeable [34], and vitamin D supplementation was commonly used in the form of vitamin D2, possibly for reasons of being able to treat a wider range of conditions (studies have claimed that vitamin D2 lowers the incidence of falls and non-vertebral fractures compared with vitamin D3) [31], for example, in North America [28]. However, with 25(OH)D as a definitive indicator of vitamin D status, vitamin D3 is gradually being found to be a more efficient form to utilize [35]. Of course, the ability of vitamin D3 to be utilized effectively may depend on the frequency of dosage administration, with a study by Laura Tripkovic et al. Noting that the response to vitamin D3 was significant when it was given in a bolus dose, but lost its effect with daily supplementation [36]. Thus, the significant effectiveness of vitamin D2 supplementation may benefit from its wider use, the nonsignificant effectiveness of vitamin D3 supplementation may be influenced by the frequency of dosage administration, and the overall nonsignificant effectiveness result is influenced by a combination of these 2 factors superimposed on the unequal number of studies (1 studies of vitamin D2 supplementation and 3 study of vitamin D3 supplementation); Differences between different populations and the aggregate may need to be explained at the genetic level. Psoriasis Risk Associated with Serum 25(OH)D Levels [37]. VDR (vitamin D receptor) is responsible for mediating 25(OH)D synthesis, inducing proliferation and differentiation of human keratinocytes, and regulating the immune system. It has more than 200 single base polymorphisms (SNPs), commonly associated with psoriasis [38–40]. Several population-specific studies have shown that VDR polymorphisms are associated with psoriasis risk in Asian populations such as, for example, Koreans [41, 42], Chinese [43] or Turks [44, 45], whereas the opposite conclusion has been found in European populations such as Italians [46] and Croats [47]. Thus, the higher efficacy rates in Asian populations compared to European populations may be related to the single-base polymorphisms of the VDR, whereas the overall general result of no significant efficacy rate is due to the limited number of studies and their uneven distribution (3 studies in Europe and 1 study in Asia).

Third, the use of markers to compensate for the limitations of evaluation metrics. There are various evaluation metrics for psoriasis, but current efficacy measures may lack validity, reliability, sensitivity to change, and feasibility [48, 49]. Therefore, given the association between CRP and psoriasis, we used CRP as a marker in an attempt to validate weak validity not reflected by efficacy indicators [29]. The results showed that although this weak validity was numerically reflected, it was not statistically supported [21]. This gives us an indication that the continued search for and utilization of some strong association markers may be able to fill the gaps in the existing measures and provide stronger evidence of effectiveness.

Of course, there are still some limitations in present study, and the possible reasons are as followed: 1. Relatively small number of studies and cases eligible for inclusion, with some studies having missing data results or cases missing visits/withdrawals; 2. The included studies differed in terms of the daily or total dose used, and the mode of intake. 3. Studies focused too little on adverse events, with only 1 of the 4 double-blind RCTs included in the study reporting adverse events. Therefore, the results of the current meta-analysis should be interpreted with caution, taking into account other potential confounding factors.

In summary, despite the aforementioned shortcomings of this study, we report a latest meta-analysis, The results showed that, in terms of effectiveness, vitamin D supplementation failed to have a significant impact on the overall effectiveness evaluation of the PASI, DLQI, PDI and CRP, and therefore we are in agreement with the findings of Formisano [11] and Theodoridis et al. [12] The difference is that in the subgroup analysis, vitamin D2 seems to be more effective than vitamin D3, and Asians seem to be more effective than Europeans, an information that should not be ignored; in terms of safety, no serious adverse effects were found, except for minor discomfort in very few cases, but we need to be cautious in our evaluation as the optimal dosage still needs to be explored. Nevertheless, in order to further investigate the efficacy and safety of vitamin D supplementation, we call for more well-designed, large-scale, prospective randomized studies in terms of optimal dosage, different populations, and different vitamin D types.

## Conclusion

The combined analyses suggest that although the efficacy of vitamin D supplementation in psoriasis is not significant and the safety profile needs to be explored, the information that the use of vitamin D2 or the possibility of better outcomes in Asian populations inspires the need for more high-quality studies to further explore the possible potential effectiveness. At the same time, the search for a new prognostic index that combines clinical and laboratory factors should be pursued to compensate for the shortcomings of existing measures and provide stronger evidence of efficacy.

## Supporting information

S1 Table PRISMA 2020 checklist. (PDF)

S2 Table Detailed search strategy in four databases. (DOCX)
