# Supplementary material for: Efficacy and safety of vitamin D supplementation on psoriasis: A systematic review and meta-analysis
Source: PLoS One. 2023 Nov 15;18(11):e0294239. doi: 10.1371/journal.pone.0294239 (PMC10650996; doi:10.1371/journal.pone.0294239)
Supplement: S2 Table — (DOCX) [file pone.0294239.s002.docx]

| **S2 Table.** Detailed search strategy in four databases. | | |
| --- | --- | --- |
| Database | Search strategy | Search results (number) |
| Pubmed | (((Oral)OR(Supplementation))AND ("Vitamin D"[Mesh])) AND (("Psoriasis"[Mesh]) OR (((((Psoriases) OR (Pustulosis of Palms and Soles)) OR (Pustulosis Palmaris et Plantaris)) OR (Palmoplantaris Pustulosis)) OR (Pustular Psoriasis of Palms and Soles))) | 128 |
| Embase | ((Oral or Supplementation) and Vitamin D and (Psoriasis or (Psoriases or (Pustulosis of Plams and Soles) or Pustulosis Palmaris et  Plantaris or Palmoplantaris Pustulosis or (Pustular Psoriasis of Palms and Soles)))).af. | 800 |
| Web of Science | (((Oral)OR (Supplementation)) AND (Vitamin D)) AND ((Psoriasis) OR (((((Psoriases) OR (Pustulosis of Palms and Soles)) OR (Pustulosis Palmaris et Plantaris)) OR (Palmoplantaris Pustulosis)) OR (Pustular Psoriasis of Palms and Soles))) (Topic) and Preprint Citation Index (Exclude – Database) | 638 |
| Cochrane | ((Oral or Supplementation) and Vitamin D and (Psoriasis or (Psoriases or (Pustulosis of Plams and Soles) or Pustulosis Palmaris et  Plantaris or Palmoplantaris Pustulosis or (Pustular Psoriasis of Palms and Soles)))).af. | 69 |
